# Green HPLC technique development for the simultaneous determination of the potential combination of Mirabegron and Tamsulosin

**DOI:** 10.1038/s41598-025-89216-5

**Published:** 2025-02-27

**Authors:** Eman A. Bahgat, Hanaa Saleh, Islam M. Darwish, Omar M El-Abassy

**Affiliations:** 1 Pharmaceutical Analytical Chemistry Department, Faculty of Pharmacy, Zagazig University, Zagazig, 44519 Egypt; 2 Egyptian Drug Authority, Giza, 12622 Egypt; 3 Pharmaceutical Chemistry Department, Faculty of Pharmacy, Egyptian Russian University, Badr City, Cairo Egypt

**Keywords:** Mirabegron, Tamsulosin, HPLC, AGREE, BAGI, Interval plot, Chemistry, Analytical chemistry

## Abstract

Mirabegron and tamsulosin have recently been prescribed to men with overactive bladder for the treatment of benign prostatic hypertrophy. An efficient and environmentally friendly HPLC method was developed to accurately measure the levels of mirabegron and tamsulosin in both their pure form and in medication formulations. Full separation was achieved on an X-Bridge C18 column using a gradient elution of (The A mobile phase was a buffer solution containing 1 mL of trifluoroacetic acid and 3 mL of triethylamine in 1,000 milliliters of water, the pH of the solution was then adjusted to 3 using triethylamine and the B-mobile phase was acetonitrile). The chromatographic peaks were obtained at a wavelength of 220 nm. Mirabegron and tamsulosin were identified with retention time values of 2.4 min and 8.9 min, respectively. In the concentration ranges of 2.5–55 µg/mL for mirabegron and 5–110 µg/mL for tamsulosin, remarkable linearity was seen. The limits of detection for the two analytes were 0.28 and 0.55 µg/mL, respectively, and their R^2^ values were 0.9999. The new HPLC method was evaluated for its environmental friendliness using the Analytical GREEness (AGREE) metric. Furthermore, the suggested technique was considered practicable based on the evaluation conducted using the Blue Applicability Grade Index (BAGI) assessment. Both evaluation methods were quite successful, yielding scores of 0.52 and 80, respectively. Compared to the TLC-reported method, HPLC is the preferred choice for the separation of the two analytes due to its sensitivity.

## Introduction

Overactive bladder syndrome (OAB) is a type of storage lower urinary tract symptoms (LUTS) that includes urgent need to go to the bathroom, with or without incontinence, often happening at night^1^. Overactive bladder syndrome is a persistent medical problem that affects individuals of both genders, resulting in a significant decline in their overall quality of life^2,3^. Although the occurrence of this ailment is more common among those aged 40 and beyond, it may affect both children and young people^4^. According to research conducted in 2011 and using a sample of 10,000 people from Europe, it was shown that about 36% of males and 43% of females aged 40 and above had symptoms indicative of OAB^5^. According to the findings of the National Overactive Bladder Evaluation (NOBLE) program, the prevalence of approximately 33 million persons with overactive bladder (OAB) in the United States population was observed. The OAB has a substantial impact on the economy. For example, a study conducted in the United States in 2005 found that the yearly costs incurred by individuals with OAB surpassed 12 billion USD. These expenditures include indirect costs, such as the temporary impairment of work performance^6^. The dominant pharmaceutical treatment for LUTS in males with benign prostatic hyperplasia (BPH) is the alpha-1-adrenoceptor blocker (A1B). Nevertheless, even receiving A1B therapy, signs of OAB may continue to exist^7^. The guideline established by the European Urologic Association in 2015 suggests that antimuscarinics or beta-3-adrenoceptor agonists (B3A) should be used in men with benign prostatic hyperplasia (BPH) for the treatment of moderate-to-severe lower urinary tract symptoms characterized by predominant bladder storage symptoms. In cases where both monotherapies prove inadequate in alleviating symptoms, a combination therapy is recommended^8^.

**Fig. 1:**
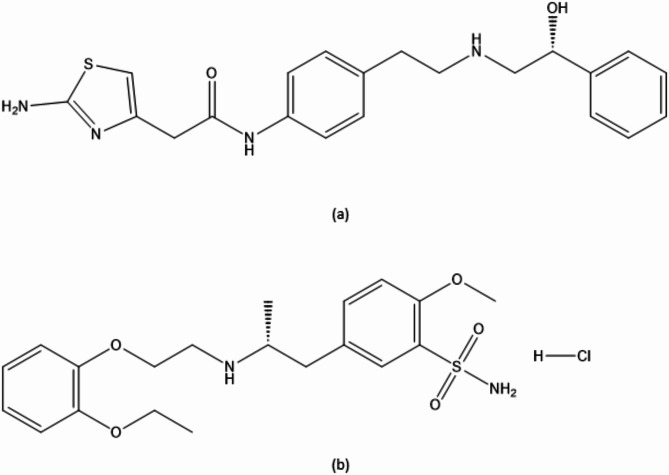
Chemical structure of (**a**) MIR (**b**) TAM.

Mirabegron (MIR) is a recently approved medicine by the FDA for the treatment of idiopathic overactive bladder^9^. Mirabegron functions as a β_3_ specific receptor agonist, exerting its effects through the relaxation of the detrusor muscle. Regarding the chemical composition of the compound, MIR can be described as (N-[4-[2-[[(2R)−2-hydroxy-2-phenylethyl] amino] ethyl] phenyl] acetamide)^10^(Fig. 1a). Tamsulosin (TAM), is an α1-adrenoceptor antagonist with the chemical formula 5-[(2R)−2-[2-(2-ethoxyphenoxy)ethylamino]propyl]l.2-methoxybenzene-1-sulfonamide^11^ that is employed in the management of lower urinary tract symptoms linked to benign prostatic hyperplasia (BPH) (Fig. 1b).

Multiple trials have demonstrated the efficacy and safety of MIR as an add-on therapy to TAM as a treatment for symptoms of obstruction of the bladder (OAB) caused by benign prostatic hyperplasia (BPH) in males with a minimal incidence of adverse effects^12^. Mirabegron and tamsulosin combination treatment for OAB has shown benefits in symptom relief without producing extra adverse effects in recent clinical studies. The combined treatment of tamsulosin and mirabegron is thus anticipated to be actively investigated as a substitute for traditional medications in individuals who are unable to tolerate the inadequacies of current drug regimens or adverse drug reactions^13^. Even though there have been a number of chromatographic studies done on MIR detection^14–20^and TAM determination^21–30^, the literature review revealed that there was just one single HPTLC technique documented for the quantification of MIR and TAM together^31^.

Liquid chromatography (LC) is the most often used and applied technique in the pharmaceutical industry. Furthermore, it is the most efficient and versatile method for the concurrent separation and quantitative measurement of different components found in a mixture^32^. To reduce the amount of hazardous organic solvents produced every day worldwide or to replace them with safer alternatives, it is crucial to apply the principles of green analytical chemistry (GAC) while continuing to routinely analyze pharmaceuticals using liquid chromatography (LC) for quality control (QC) without compromising performance^33^. The current research used the Analytical GREENNESS metric methodology (AGREE) to evaluate the environmental greenness of the suggested strategy^34^. The findings demonstrate that the proposed method has outstanding environmental sustainability. In addition, the newly implemented Blue Applicability Grade Index (BAGI) tool was used to evaluate the blueness of the technique^35^. The main goal of the proposed study is to use the GAC principle to make a simple, low organic consumption, sensitive and cost-effective high-performance liquid chromatography (HPLC) method for evaluating the mixture.

## Experimental

### Materials and reagents

#### Chemicals and reagents

The chemicals used in this study were of HPLC quality, including trifluoroacetic acid, triethylamine, methanol, and acetonitrile. Sigma Aldrich supplied these for use in the mobile phase and diluent production. The MIR and TAM reference standards were purchased from Sigma–Aldrich. The medications Betmiga 50 mg^®^ and Tamsul 0.4 mg^®^ were obtained from a local market. The study was carried out with Milli-Q water, which was produced internally.

### Instruments and software details

An EAB 224e Adam equinox semi-microbalance is used to measure the weight of all samples, compounds, and standards. The mobile phase buffer with a pH of 3.0 was created and then assessed using a JENWAY 3510 pH meter. The whole study was carried out using an Agilent HPLC type Alliance 1260, equipped with 2,998 Photodiode-Array Detector (PDA) detectors and 2,489 UV detectors. The development of the chromatographic technique included the use of an X-Bridge C18 column (4.6 × 150 mm, 3.5 μm) obtained from Waters.

### Stock standard solutions

Stock standard solutions were made with concentrations of 100 µg/mL MIR and 200 µg/mL TAM, respectively, by measuring and transferring 5 mg of MIR and 10 mg of TAM into 50 mL clean and dry volumetric flasks. After that, 10 ml of methanol was added. The flask was filled to the mark with distilled water. The resulting stock solution was filtered using a 0.22 μm nylon filter.

### General procedures

#### Chromatographic conditions

A buffer solution containing 1 mL of trifluoroacetic acid and 3 mL of triethylamine in 1,000 milliliters of water was used to create Phase A of the mobile phase. The pH of the solution was then adjusted to 3 using triethylamine. The B-mobile phase was acetonitrile. The process of separation was accomplished using a linear gradient procedure in the following manner: The developed gradient program started with a high proportion of the aqueous phase (80:20, A: B) for a duration of 5 min, followed by a transition to (60:40, A: B) over a period of 4 min. After a duration of 4 min, the gradient program was reverted back to its original conditions. The rate of flow was one milliliter per minute. The injection volume was 10 µl. The total duration of the experiment was 13 min, with a wavelength of 220 nm.

#### Linearity

The linearity of the samples was assessed by systematically diluting each analyte from the respective stock standard solutions individually. This process resulted in solutions with concentrations ranging from 2.5 to 55.0 µg/mL of MIR and 5.0 to 110.0 µg/mL of TAM. The mobile phase was used as the solvent for this dilution. Injecting the solutions in triplicate allowed us to obtain chromatograms under the chromatographic conditions mentioned previously. We plotted the average peak area against the relevant concentrations on both the integrated and calibration plots.

#### Analysis of dosage forms

The Betmiga^®^ dosage form had ten tablets that were accurately weighed, crushed, and mixed. An amount of powder equal to one tablet was transferred to a 100 mL volumetric flask. Twenty Tamslu^®^ capsules were properly measured, and their average weights were calculated. The capsule contents were well mixed before being transferred to a 25 mL volumetric flask at a specific weight comparable to 5 mg. The volume of each flask was then adjusted to 30 mL and 15 mL respectively with methanol. An ultrasonic shaker was used to stir the flasks for 15 min. After filtering using a 0.45-µm membrane filter, the contents of the flasks were further diluted. To create a solution including MIR and TAM, aliquots of Betmiga^®^ and Tamsul^®^ filtrates were placed in a 25-mL volumetric flask and diluted with the mobile phase.

## Results and discussion

### Optimization of chromatographic conditions

It is not practicable to simultaneously determine MIR and TAM using zero-order simple UV spectrophotometry. A novel technique was devised to analyze mixes of MIR-TAM using a gradient liquid chromatographic method combined with diode array detection (Table 1). This method offers a viable approach for conducting regular quality control examinations. The primary objective in the development of LC methods is to attain adequate resolution with satisfactory peak symmetry within a tolerable analytical timeframe. This objective was accomplished by performing many experiments aimed at optimizing the stationary and mobile phases. In order to optimize the stationary phase, two columns were tested: the X-Bridge C18 column with dimensions of 4.6 × 150 mm, 3.5 μm and the X-Bridge C18 column with dimensions of 4.6 × 250 mm, 5 μm. The X-Bridge C18 column, size 4.6 × 150 mm x 3.5 μm, was determined to be optimal due to its ability to achieve the best retention period for the two medicines. Different volumes of various compositions of mobile phases were subjected to testing. A mixture of 0.001% trifluoroacetic acid solution, triethylamine and acetonitrile was found to be the most effective mobile phase. An experiment was conducted using orthophosphoric acid, which led to the occurrence of a tailing in the peaks. Additionally, octansulphonate and heptansulphonte were used, resulting in the occurrence of peaks overlap. In addition, the use of a mobile phase containing a significant amount of acetonitrile in an isocratic manner led to a complication in the MIR peak. Also, there was a lack of retention and clear co-elution between the two peaks. In order to solve these issues and achieve a perfect resolution between MIR and TAM, a gradient elution technique was used, initiating with a low amount of acetonitrile.

**Table 1:** HPLC gradient elution program.

Time (min)	Mobile Phase A	Mobile Phase B
0	80	20
5	80	20
9	60	40
13	80	20

### Method validation

The process of validating the proposed approach was conducted in accordance with the principles set out by the ICH^36^.

#### System suitability

Table 2 displays the computed system suitability characteristics, namely retention time, theoretical plates, tailing factor, resolution, and selectivity.

**Table 2:** Validation criteria and system suitability for HPLC analysis of MIR and TAM.

Parameter	MIR	TAM
Retention Time (t_R_)	2.401±0.03	8.901±0.03
Theoretical Plates (N)	5126	31,612
Resolution (Rs)		38
Tailing factor	1.38	1.30
Selectivity		27.95
Linearity range (µg/mL)	2.5–55	5–110
Intercept	19.96	54.72
S.D. of intercept	8.67	12.84
Slope	100.86	75.82
Determination coefficient (R^2^)	0.9999	0.9999
LOD^*^ (µg/mL)	0.28	0.55
LOQ^*^ (µg/mL)	0.86	1.69
Accuracy	100.50±0.63	98.95±1.10

*LOD = 3.3 × SD of intercept/slope, LOQ = 10 x Sd of intercept/slope.

#### The method selectivity

**Fig. 2:**
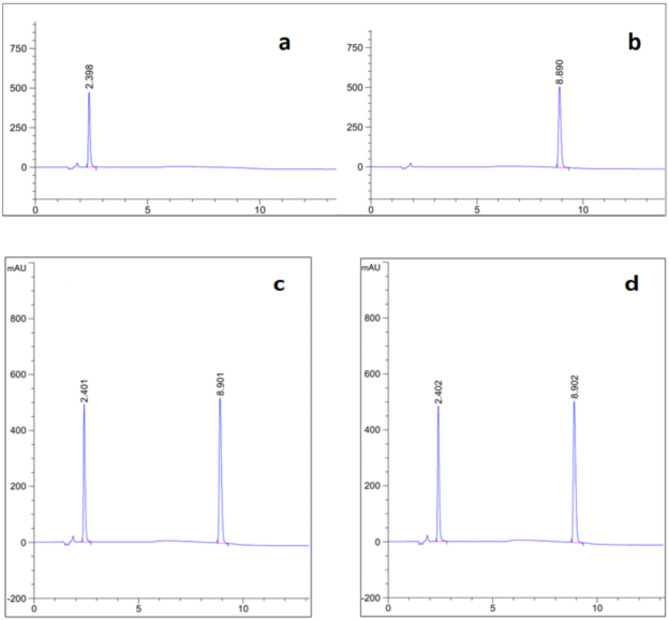
(**a**) MIR 25 µg/mL and (**b**) TAM 50 µg/mL, chromatogram of 25 µg/mL MIR and 50 µg/mL TAM in (**c**) bulk powder and (**d**) dosage forms (Betmiga and Tamslu).

The comparison of chromatograms between the raw analytes and those obtained from dosage forms (Fig. 2) indicated the absence of any interference caused by commonly used excipients in tablets and capsules.

#### Linearity and range

The correlation between peak area and concentration was shown to be linear for MIR and TAM within the concentration ranges of 2.5–55 and 5–110 µg/mL, respectively. The results are presented in Table 2.

#### LOQ and LOD

The slope of the relative standard calibration curves and standard deviations of the intercept were used to calculate the limits of detection (LOD) and quantitation (LOQ) of both medications (Table 2).

#### Accuracy and precision

The accuracy of the measurements was effectively determined by calculating the percentage of recovered substance in the three replicates of the three distinct concentrations falling within the linear range for MIR and TAM, as shown in Table 2. The different three concentration levels were 5, 25, and 50 µg/mL, 10, 50, and 100 µg/mL for MIR and TAM, respectively. According to Table 3, the suggested approach is precise. The study aimed to assess the repeatability precision by administering standard solutions at three different concentration levels, three times daily. The process was replicated on three separate days to evaluate the intermediate level of precision. The different three concentration levels for repeatability and intermediate precision were 5, 25, and 50 µg/mL, 10, 50, and 100 µg/mL for MIR and TAM, respectively.

**Table 3:** Repeatability and intermediate precision results for the simultaneous determination of MIR and TAM in pure form using the proposed HPLC method.

Drug	Repeatability			Intermediate precision		
MIR	**Conc. taken (µg/mL)**	**Mean ± SD**	**RSD %**	**Conc. taken (µg/mL)**	**Mean ± SD**	**RSD %**
	5	100.405 ± 0.232	0.231	5	100.138±0.524	0.523
	25	100.278±0.082	0.082	25	100.463±0.308	0.306
	50	100.542±0.099	0.099	50	100.509±0.189	0.188
TAM	10	99.383±0.131	0.132	10	99.304±0.331	0.334
	50	100.101±0.084	0.084	50	100.840±0.793	0.786
	100	100.410±0.159	0.159	100	100.357±0.079	0.078

#### Chromatographic method robustness

In order to demonstrate the durability of the suggested approach, minor modifications were made to the chromatographic components, and the impact on certain chromatographic parameters was noted (Table 4). The findings of the study suggest that the approach exhibits robustness.

**Table 4:** Chromatographic Method Robustness.

Drugs	Factor	Analyst to analyst		Column temperature ^o^C		Flow rate (mL/min)	
	Changes			24,25,26		0.95,1,1.05	
	Tested parameters	Peak area	Retention time	Peak area	Retention time	Peak area	Retention time
MIR	RSD%	1.824	0.362	1.453	0.214	1.721	0.742
TAM	RSD%	1.963	0.472	1.683	0.162	1.824	0.947

#### Application to pharmaceutical formulations

The application of the suggested research was used for different dosage forms MIR in tablets and TAM in capsules. Regardless, there is a newly published HPTLC technique for determining the binary mixture^31^. The suggested research exhibits many advantages in various aspects. The proposed research exhibits greater sensitivity for MIR and TAM. Also the analysis conducted on MIR in tablets and TAM in capsules yielded recovery rates of 100.11% and 99.53%, respectively (Table 5).

**Table 5:** Statistical evaluation of proposed and published techniques for analysis of pharmaceutical dosage forms containing MIR and TAM.

Parameters	Proposed Method		Published Method [31]	
	MIR	TAM	MIR	TAM
Mean^a^	100.11	99.53	99.67	99.84
SD	0.52	0.48	0.68	0.90
Student’s *t*-test	0.89(2.78)^*^	0.53(2.78)^*^		
F-value	1.71(19.00)^*^	3.52(19.00)^*^		

^a^Average of three measurements.

^*^The parentheses indicate the theoretical values of t and F at *P* = 0.05.

**Fig. 3:**
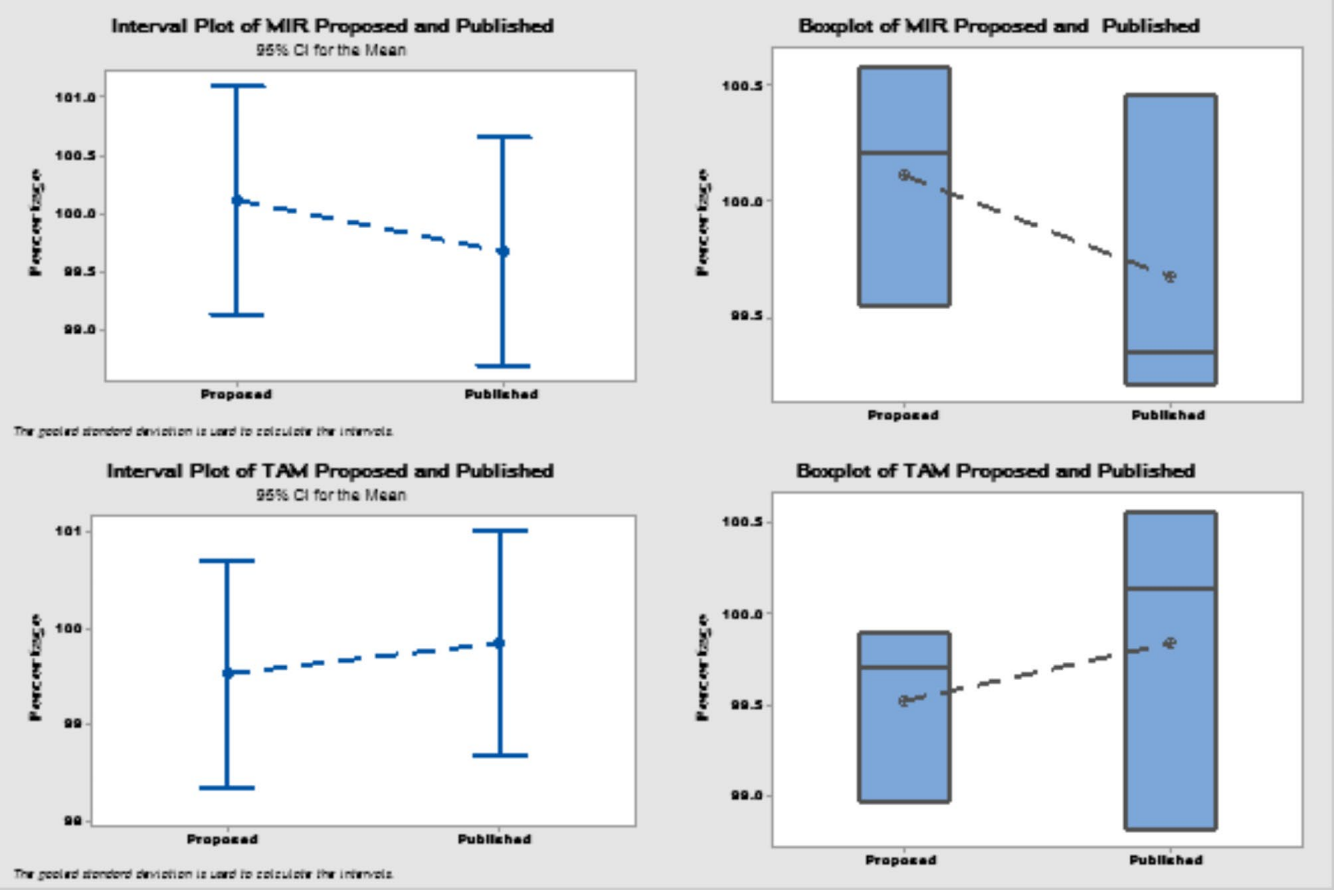
Interval and boxplot plot of the proposed and published methods.

The statistical comparison between the proposed approach and the published one was carried out using Student’s t-test and F-test. According to Table 6, there was no discernible difference between the two approaches. Additionally, the interval plot test was used. The vertical lines representing confidence intervals on plots imply that the interval mean is located at their midpoint. According to these graphs, the group intervals are not significantly different from one another (Fig. 3). The Boxplot is another useful tool for data visualization since it depicts the distribution of data across multiple categories. It displays boxplots of the proposed methods. The interquartile range is shown by the middle box, which includes a line showing the median, upper lines representing higher values, and whiskers signifying lower values. The boxplot depicts the distribution of data within each group. (Fig. 3)

### Assessment of ecological effect and comparison to the previously documented analytical technique

While developing analytical techniques, it became crucial to evaluate their ecological effect to provide a clear and objective evaluation that could be used to compare proposed and reported approaches in the future^31^. AGREE offers a clock-shaped graph that is split into 12 pieces, representing the twelve fundamentals of Green Analytical Chemistry^37–39^. The suggested and reported techniques^31^ were evaluated using the AGREE tool. Table 6presents a comprehensive comparison between the suggested methodology and previously published methods that included real data. The two approaches indicate the presence of a red zone (3) on the AGREE scale. A red zone has emerged as a result of the offline sampling and transportation to quality control (QC) labs, which is essential owing to the separation between pharmaceutical manufacturing and QC locations. The suggested LC method demonstrated advantages over the other documented chromatographic methodology in terms of the number of stages, analysis duration, and the usage of significant amounts of organic solvents (Table 6). The proposal introduces a new measure called the Blue Applicability Grade Index (BAGI) to evaluate the practicability of an analytical method^40–42^. When used in conjunction with the well-known green metrics, the BAGI framework shines a light on the practical aspects of White Analytical Chemistry. The color gradient of the pictogram indicates the extent to which the method conforms to the defined criteria. If the method wants to be called “practical,” it has to get 60 or more points Table 6.

**Table 6:**
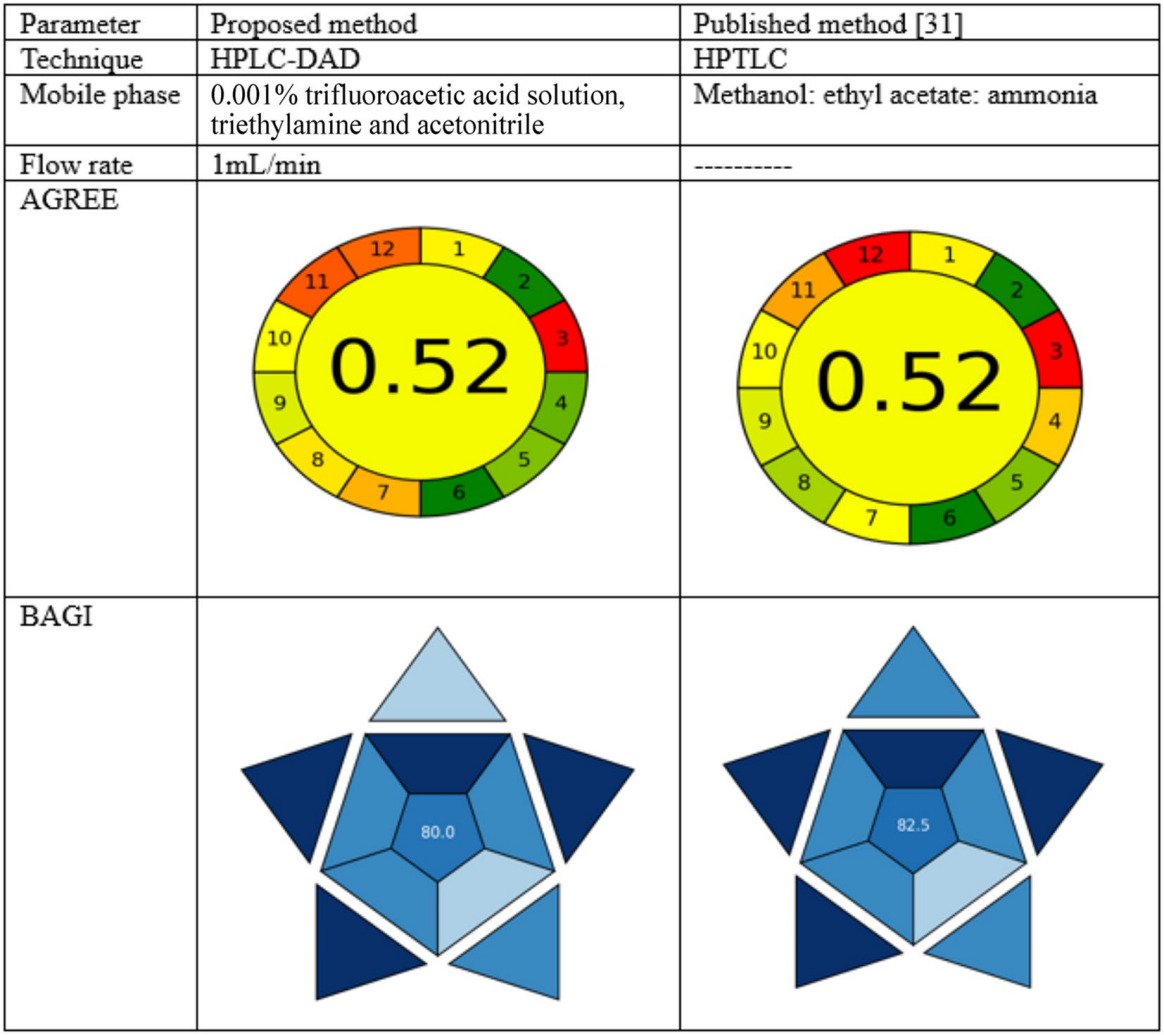
A comparative study of the proposed and published methods for MIR and TAM determination

## Conclusion

A novel and cost-efficient technique was shown for the concurrent HPLC analysis of mirabegron and tamsulosin in pharmaceutical formulations, ensuring accuracy and precision. Moreover, a comparative analysis was conducted between the suggested methodology and previously documented approaches, which revealed no discernible difference between the two approaches. The evaluation of the greenness using the AGREE metric tool included a comparison with the previously published method. The assessment of the Blue Applicability Grade Index methodology further shows that the recently proposed method is practicable. The proposed technique was validated using the rules set by the International Council for Harmonization.

## Data Availability

All data generated or analysed during this study are included in this published article.
