# Publisher Correction: Green HPLC technique development for the simultaneous determination of the potential combination of Mirabegron and Tamsulosin

**DOI:** 10.1038/s41598-025-98289-1

**Published:** 2025-04-24

**Authors:** Eman A. Bahgat, Hanaa Saleh, Islam M. Darwish, Omar M El-Abassy

**Affiliations:** 1 Pharmaceutical Analytical Chemistry Department, Faculty of Pharmacy, Zagazig University, Zagazig, 44519 Egypt; 2 Egyptian Drug Authority, Giza, 12622 Egypt; 3 Pharmaceutical Chemistry Department, Faculty of Pharmacy, Egyptian Russian University, Badr City, Cairo Egypt

Publisher Correction: *Scientific Reports* 10.1038/s41598-025-89216-5, published online 27 February 2025

In the original version of this Article authors Islam M. Darwish and Omar M El-Abassy were incorrectly affiliated. Their correct affiliations are listed below.

Author Islam M. Darwish is affiliated with “Egyptian Drug Authority, Giza, 12622, Egypt” and author Omar M El-Abassy is affiliated with “Pharmaceutical Chemistry Department, Faculty of Pharmacy, Egyptian Russian University, Badr City, Cairo, Egypt”.

The original Article has been corrected.

